# Psychosocial Impact of Pediatric and Adolescent Hyperhidrosis: A Systematic Review and Call for Research

**DOI:** 10.1111/jocd.70213

**Published:** 2025-04-28

**Authors:** Sheila Sharifi, Mohammad Jafferany

**Affiliations:** ^1^ Dr. Phillip Frost Department of Dermatology and Cutaneous Surgery University of Miami Miller School of Medicine Miami Florida USA; ^2^ Department of Psychiatry and Behavioral Sciences Central Michigan University College of Medicine Mount Pleasant Michigan USA

**Keywords:** pediatric, primary hyperhidrosis, psychodermatology, psychosocial

To the Editor,

Hyperhidrosis is characterized by excessive sweating of the axilla, palms, soles, and face, estimated to affect 3% of the United States population [1]. The disease extends beyond physical impairment, significantly impacting daily functioning and psychosocial well‐being. The onset of primary hyperhidrosis is thought to be during the adolescent years or earlier, although studies examining the psychological impacts of the disease in pediatric and adolescent populations remain limited [1]. This systematic review is the first to synthesize and evaluate the literature on the psychosocial effects of primary hyperhidrosis in these populations.

A search of the PubMed, EMBASE, and Web of Science databases was conducted using the search terms “pediatric hyperhidrosis” or “childhood hyperhidrosis” or “adolescent hyperhidrosis” and “psychosocial” or “mental health” or “psychological” or “emotional” or “social impact” or “quality of life” or “self‐esteem” or “anxiety” or “depression” or “well‐being” or “social functioning.” After removal of duplicate articles, our search yielded 277 studies for screening. Studies were included if they discussed the appropriate topic, were the correct study type (e.g., clinical trials, cohort studies, case–control studies, cross‐sectional studies, case reports, case series, or interview studies), and were published in peer‐reviewed journals in English. Exclusion criteria consisted of studies irrelevant to the topic, incorrect publication types (i.e., review articles, commentaries, protocols, or letters to the editor), and publications in languages other than English. Ultimately, 12 studies were included in our review, from which data was extracted and synthesized. Quality assessment included validating study methodologies, population characteristics and any diagnostic tools used for psychological diagnoses. A PRISMA diagram is outlined in Figure 1, with detailed findings of each study in Table 1.

**FIGURE 1:**
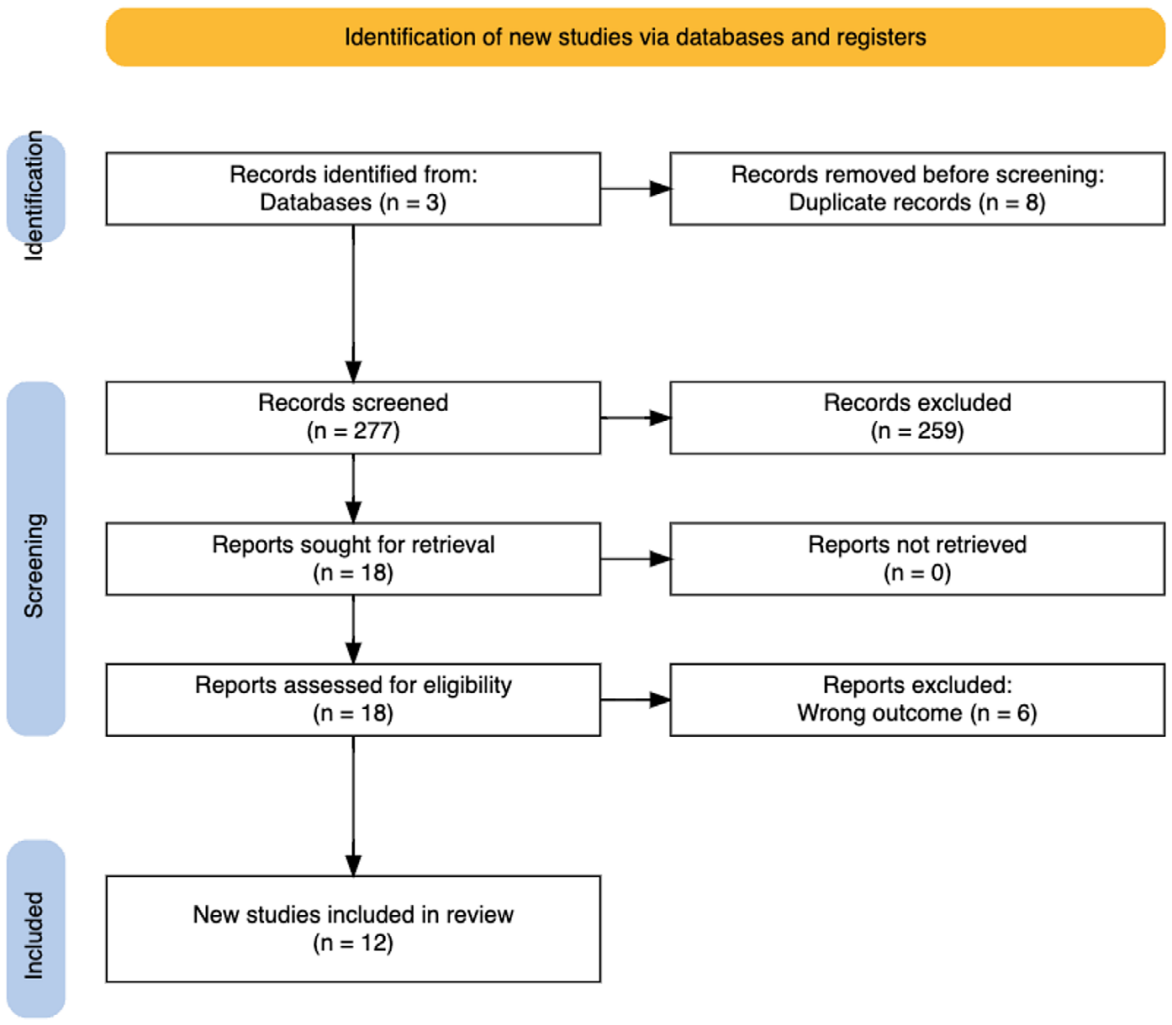
PRISMA Diagram.

**TABLE 1:** Characteristics of included studies.

Study/year	Type of study/participants	Key findings
De Bie et al. 2023	Retrospective cohort study of 58 pediatric patients	Patients reported poor social functioning at baseline (9.5 +/− 3.1 out of 25), poor personal interaction with others (11.1 +/− 3.1 out of 20), and overall stress (8.1 +/− 3.2 out of 25)—Following T3 bilateral thoracoscopic sympathectomy, all QOL measures mentioned reached statistically significant differences in the > 5‐year follow‐up (*p* < 0.0001)
Glaser et al. 2015	Multicenter, nonrandomized, open‐label study of 121 adolescent patients	50.7% of patients reported feeling moderately to extremely damaged or injured emotionally because of hyperhidrosis at baseline, which decreased to 13.6% and 11.2% at 4‐ and 8‐weeks following treatment with OnabotulinumtoxinA, respectively (*p* < 0.001)
Hebert et al. 2019	Post hoc analysis of two randomized, controlled trials of 44 pediatric patients	At baseline, children < 16 years had a mean CDLQI score of 8.5 +/− 5.6, indicating moderate effect—Following treatment with 4 weeks of topical glycopyrronium tosylate, CDLQI scores improved to 9.9 +/− 5.5 (p‐values not calculated)
Kouris et al. 2015	Nonrandomized, noncontrolled clinical trial of 35 pediatric patients	Before treatment with botulinum toxin type A, mean CDLQI score was 14.65 +/− 2.35, indicating severe effect, and social isolation score* was 45.68 +/− 4.90, indicating moderate loneliness—After treatment, mean CDLQI score was 4.20 +/− 1.41 and social isolation score was 28.94 +/− 3.82 (*p* < 0.001 for both) *Using the UCLA 20‐item social isolation and loneliness questionnaire
Lima et al. 2023	Prospective cohort study of 220 pediatric and adolescent patients	Prior to treatment with ETS, 64.1% patients considered their QOL secondary to primary hyperhidrosis very poor (*p* = 0.02) with constraints on psychological welfare, study, social, and work‐related activities—24 months after surgery, QOL was significantly improved in 96.4% of patients (*p* < 0.01)
Mirkovic et al. 2018	Retrospective cohort study of 323 pediatric and adolescent patients	The median total score of the DLQI before treatment with botulinum toxin A for children aged 16–17 years was 11 (8–15), indicating severe effect, and for children younger than 16 years the total score was 12 (7–15), indicating moderate effect, with documented effects of negative self‐esteem, inhibited physical and social contacts, problems with clothing, and practical impact on schoolwork
Neves et al. 2012	Retrospective cohort study of 45 pediatric patients	Baseline mean QOL scores were similar among control and treatment groups, 84 +/− 9 and 88 +/− 8, respectively, indicating poor or very poor QOL—76.7% of patients who underwent VATS reported much better improvement in QOL, compared to 13.3% of control (*p* < 0.001)
Paller et al. 2024	Cross‐sectional study of 1671 pediatric and adolescent patients	Hyperhidrosis had moderate mean stigma scores (47.9; moderate range 45–55)—Children with hyperhidrosis had the highest percentages of T scores for depression (40.9%) and anxiety (31.8%); depression and anxiety scores were weakly correlated to stigma (depression, *r* = 0.31; anxiety, *r* = 0.34)
Paller et al. 2012	Retrospective chart review of 159 pediatric patients	Patients and parents reported social discomfort, embarrassment in social settings, and trouble in school and extracurricular activities
Trettin et al. 2022	Interview study of 10 adolescent patients	Patients reported difficulty with tasks in school and leisurely activities, feelings of despair and disappointment, deteriorated self‐esteem, withdrawal from social activities, and desires to express their burdens
Wolosker et al. 2014	Prospective cohort study of 45 pediatric patients	Before treatment, all children had poor or very poor QOL with a median score of 73—After 6 weeks of treatment with oxybutynin, 80% of the children reported improvement in QOL rated as much or slightly better with a median score of 36 (*p* < 0.001)
Wolosker et al. 2015	Prospective cohort study of 88 pediatric patients	Baseline QOL was very poor (50.1%) or poor (49.9%) in all participants—After 6 weeks of treatment with oxybutynin, QOL was slightly better (44.1%) or much better (50.8%) in all participants

Abbreviations: CDLQI: Children's Dermatology Life Quality Index; DLQI: Dermatology Life Quality Index; ETS: Endoscopic thoracic sympathectomy; QOL: Quality of life; VATS: Video‐assisted thoracic sympathectomy.

Our results highlight that the majority of pediatric and adolescent patients with primary hyperhidrosis report severe impacts on quality of life and emotional well‐being [2, 3]. Specific symptoms include poor interpersonal functioning, personal isolation and loneliness, issues with work or school‐related tasks, perceived stigma, and high social visibility [3, 4, 5]. A cross‐sectional study of 1671 pediatric and adolescent patients with chronic skin conditions found that children with hyperhidrosis had the highest rates of depression (40.9%) and anxiety (31.8%) [4].

Studies have examined the psychosocial benefits of treatment options, including anticholinergics, neurotoxins, and surgery. Within a cohort of 121 adolescents, Glaser et al. demonstrated that OnabotulinumtoxinA significantly improved emotional distress (*p* < 0.001) [2]. Thoracic sympathectomy, typically reserved for refractory disease, also enhanced psychosocial functioning. In Lima et al.'s cohort of 220 pediatric and adolescent patients, over 96% reported significant improvements in quality of life (QOL) following surgery (*p* < 0.01) [3]. Finally, Wolosker et al. administered a 6‐week course of oxybutynin to their cohort of 45 children with a significant increase in QOL scores in 80% of patients (*p* < 0.001) [6]. Importantly, none of these treatment options were associated with safety concerns in children and adolescents [2, 3, 5].

Our findings highlight the substantial psychosocial burden of primary hyperhidrosis in children and adolescents, profoundly affecting emotional well‐being, interpersonal relationships, daily functioning, and overall quality of life. Mainstay treatment options have shown promising efficacy in reducing symptoms, although further investigations with prospective designs and larger patient cohorts are needed to validate these results. Limitations of our review include small sample sizes, short follow‐up periods, and lack of standardized diagnostic tools, underscoring the need for future research. Greater collaborations between dermatology and psychiatry may aid in developing integrated treatment plans and early screening for mental health comorbidities.

## Ethics Statement

The authors have nothing to report.

## Consent

The authors have nothing to report.

## Conflicts of Interest

The authors declare no conflicts of interest.

## Data Availability

The authors have nothing to report.
